# Supplementary material for: NuRD-interacting protein ZFP296 regulates genome-wide NuRD localization and differentiation of mouse embryonic stem cells
Source: Nat Commun. 2018 Nov 2;9:4588. doi: 10.1038/s41467-018-07063-7 (PMC6214896; doi:10.1038/s41467-018-07063-7)
Supplement: Supplementary file 1 — Supplementary Information [file 41467_2018_7063_MOESM1_ESM.pdf]

Supplementary Information

NuRD-interacting protein ZFP296 regulates genome-wide NuRD localization and differentiation of mouse embryonic stem cells

Kloet et al.

Kloet et al Supplementary Figure 1

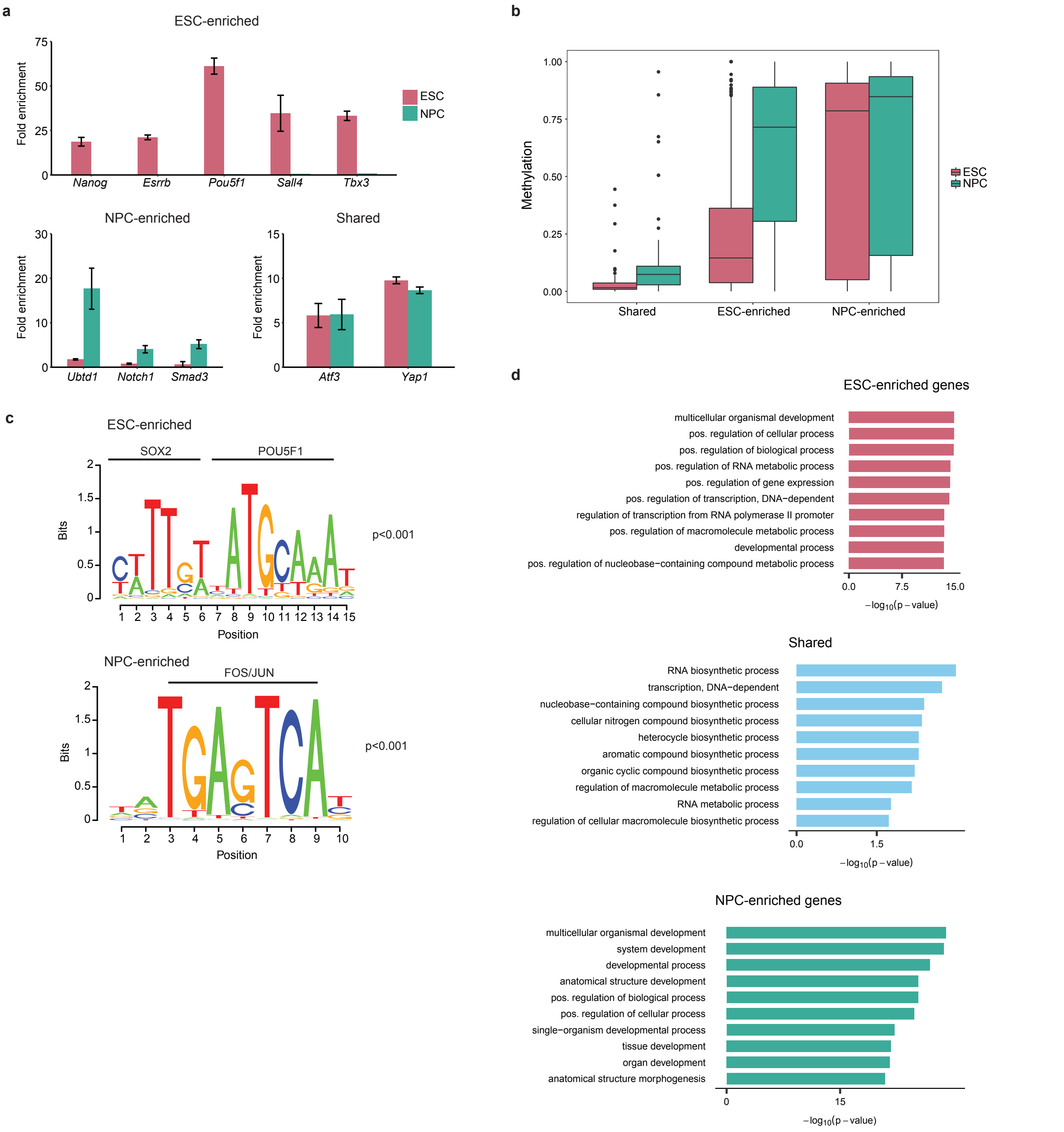

Supplementary Figure 1: Supplement to Fig. 1.
(a) ChIP-qPCR validation on MBD3 in ESCs and NPCs for ESC-enriched (top), NPC-enriched (bottom left), or shared (bottom right) NuRD binding sites. n=2 ChIPs, error bars = s.d. of technical triplicates from one experiment.
(b) The average level of DNA methylation (0 = unmethylated, 1 = fully methylated) in ESCs and NPCs (from publicly available data[56]) for shared, ESC-enriched, or NPC-enriched MBD3 ChIP-seq peaks. Box: median (central line), first and third quartile (box limits); whiskers: 1.5 × interquartile range.
(c) Most significant motif enriched under ESC-enriched (top) or NPC-enriched (bottom) MBD3 ChIP-seq peaks.
(d) Gene ontology (GO) term enrichment analysis of Biological Processes of genes within 100 kb for ESC-enriched (top), shared (middle), or NPC-enriched (bottom) NuRD ChIP-seq peaks (nearest gene only).

Kloet et al Supplementary Figure 2

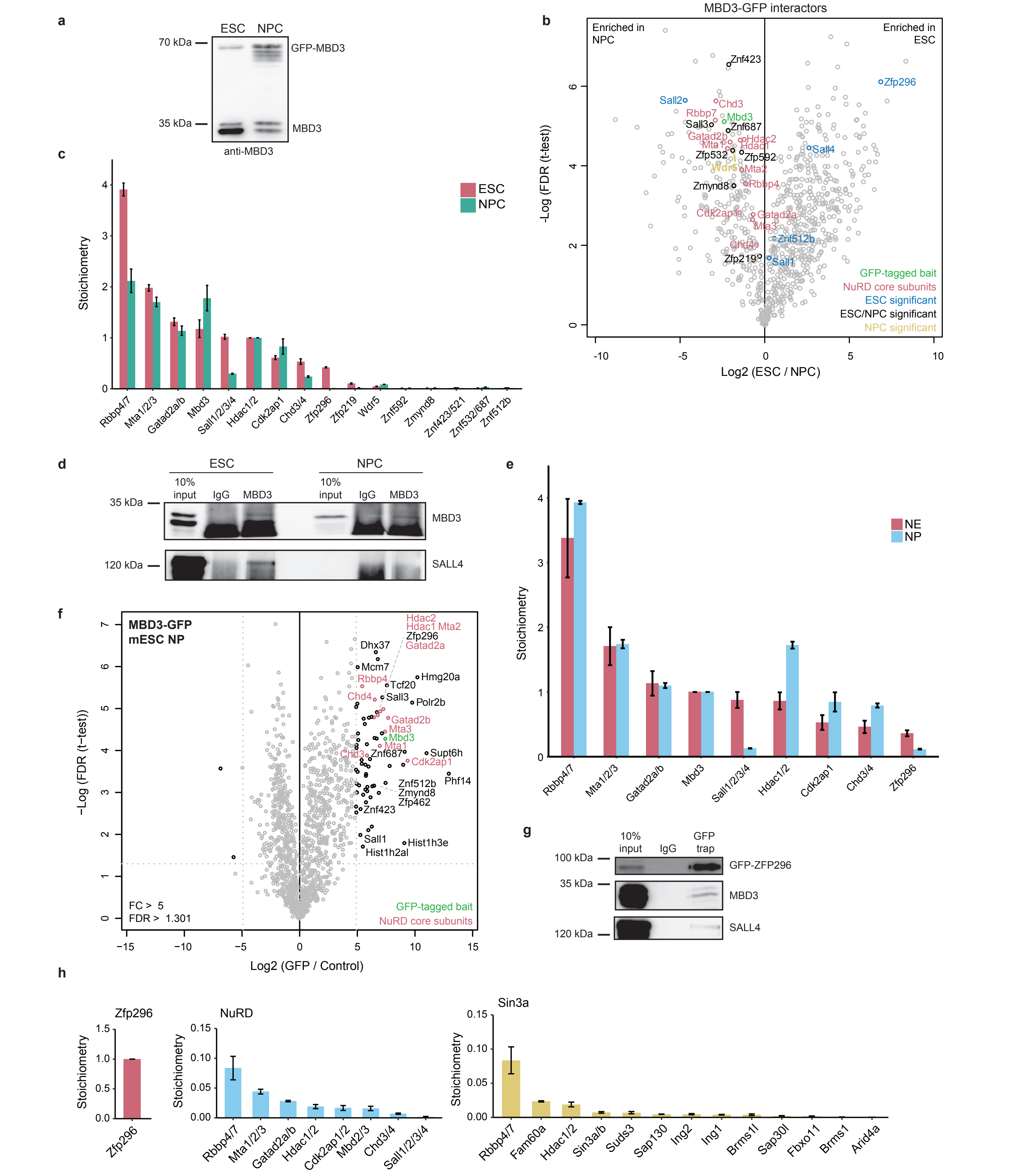

Supplementary Fig. 2: Supplement to Fig. 2.
(a) Immunoblot of endogenous and GFP-tagged MBD3 expression levels in ESCs and NPCs stably expressing the MBD3-GFP BAC.
(b) Volcano plot in which the MBD3-GFP enriched proteins from the label free GFP pulldowns in Fig. 2a,b are plotted against each other, as in Fig. 2a. In addition, proteins that were identified as significant outliers in Fig. 2a or b are colour-coded in blue or yellow, respectively, and those that were significant in both are in black.
(c) Stoichiometry of MBD3-GFP interactors in ESCs and NPCs. The iBAQ value of each protein group is divided by the iBAQ value of the core NuRD subunit HDAC1/2, then graphed with HDAC1/2 set to 1. Data are shown as mean ± s.d. (n = 3 pulldowns).
(d) Co-immunoprecipitations (Co-IP) using antibodies against MBD3 or IgG (control) on ESC and NPC nuclear extracts, followed by immunoblotting against MBD3 (top) or SALL4 (bottom).
(e) Relative abundance (stoichiometry) of MBD3-GFP interactors in nuclear extract (NE) compared to nuclear pellet (NP) fraction. The iBAQ value of each protein group is divided by the iBAQ value of the core NuRD subunit MBD3, then graphed with MBD3 set to 1. Data are shown as mean ± s.d. (n = 3 pulldowns).
(f) Volcano plot of MBD3-GFP interactors in the ESC nuclear pellet (NP) fraction. Data are graphed as in Fig. 2a.
(g) Co-IP of GFP-ZFP296 and the indicated NuRD subunits on GFP-ZFP296 ESC nuclear extracts.
(h) Stoichiometry of GFP-ZFP296 interactors in ESCs. The iBAQ value of each protein group is divided by the iBAQ value of ZFP296, then graphed with ZFP296 set to 1. Data are shown as mean ± s.d. (n = 3 pulldowns).

Kloet et al Supplementary Figure 3

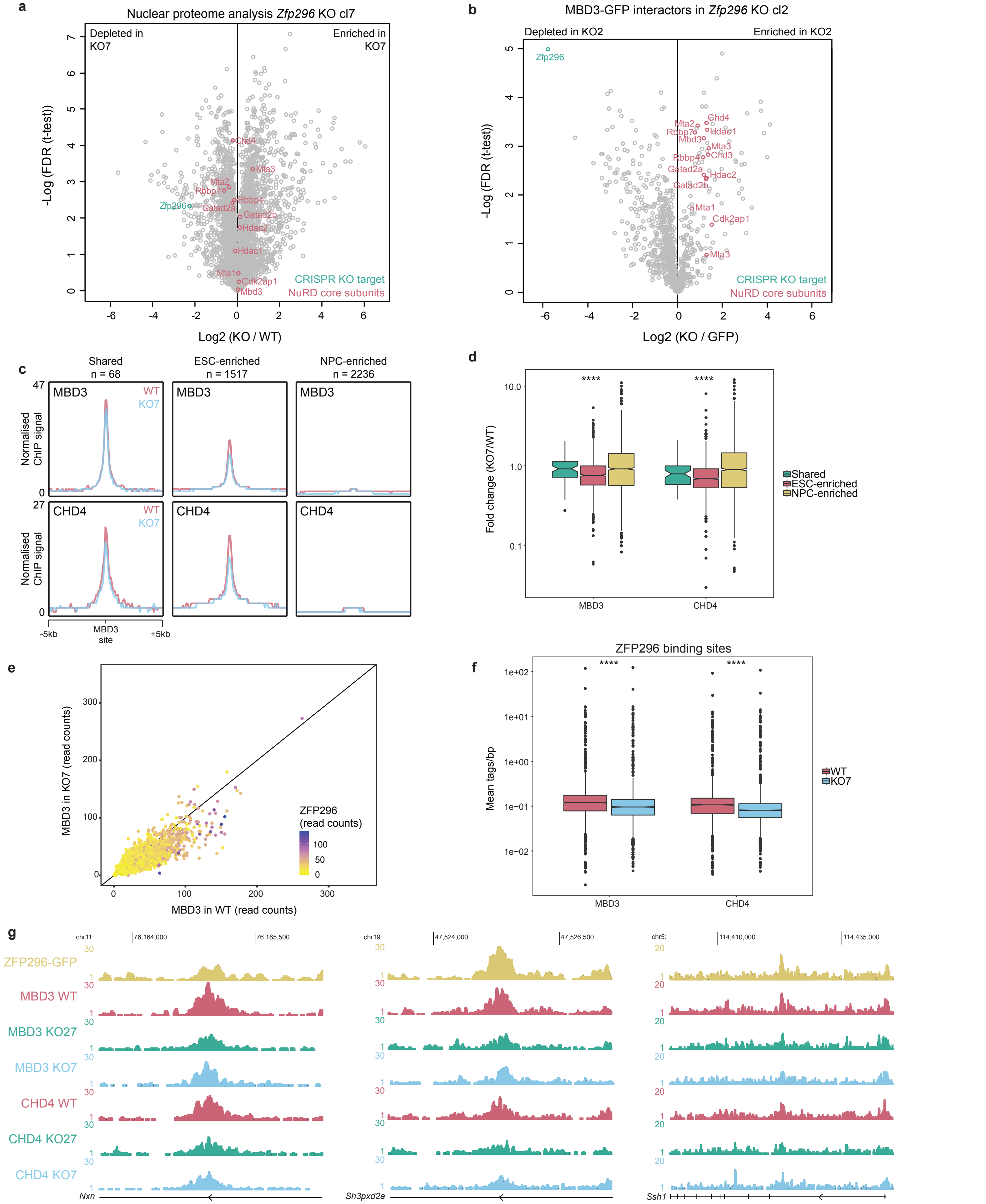

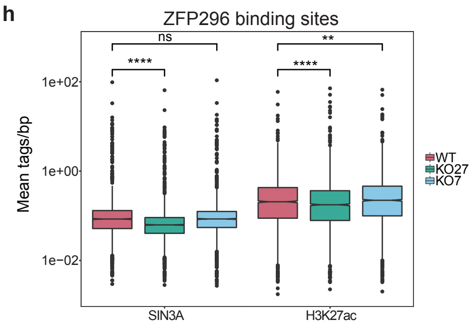

Supplementary Fig. 3: Supplement to Fig. 4.
(a) Volcano plot from label-free whole proteome mass spectrometry analysis of WT and Zfp296 KO clone 7 ESCs, graphed as in Fig. 2a. The CRISPR KO target Zfp296 is colour-coded in green, NuRD core subunits are colour-coded in red.
(b) Volcano plot of MBD3-GFP interactors from label-free GFP pulldowns on nuclear extracts from Zfp296 KO clone 2 ESCs with or without MBD3-GFP BAC. Data are plotted as in Fig. 2a. The CRISPR KO target Zfp296 is colour-coded in green, NuRD core subunits are colour-coded in red.
(c) Band plots of MBD3 and CHD4 ChIP-seq in WT and Zfp296 KO clone 7 ESCs at MBD3 binding sites for each class of NuRD binding.
(d) Fold change in ChIP-seq read counts (Zfp296 KO clone 7/WT) of MBD3 and CHD4 at MBD3 binding sites for each class of NuRD binding. Box: median (central line), first and third quartile (box limits); whiskers: 1.5 × interquartile range. **** $p < 0.0001$, as assessed by ANOVA.
(e) Scatterplot of MBD3 ChIP-seq read counts at ESC-enriched MBD3 binding sites in WT and Zfp296 KO clone 7 ESCs, coloured for the amount of ZFP296 at the same locus. The diagonal line indicates no change between the two conditions. A linear model on these data performs significantly better when taking into account ZFP296 levels as a predictive variable ($p < 0.001$, as assessed by ANOVA).
(f) Boxplots of average MBD3 and CHD4 ChIP-seq signal in WT and Zfp296 KO clone 7 ESCs at all GFP-ZFP296 peaks. Box: median (central line), first and third quartile (box limits); whiskers: 1.5 × interquartile range. **** $p < 0.0001$, as assessed by Kruskal-Wallis test.
(g) UCSC genome browser screenshots of example ZFP296-target loci where a modest decrease in NuRD binding upon Zfp296 KO can be appreciated.
(h) Boxplots of average SIN3A and H3K27ac ChIP-seq signal in WT and Zfp296 KO clone 27 and clone 7 ESCs at all GFP-ZFP296 peaks. Box: median (central line), first and third quartile (box limits); whiskers: 1.5 × interquartile range. **** $p < 0.0001$, ** $p < 0.01$, ns = not significant, as assessed by Kruskal-Wallis test.

Kloet et al Supplementary Figure 4

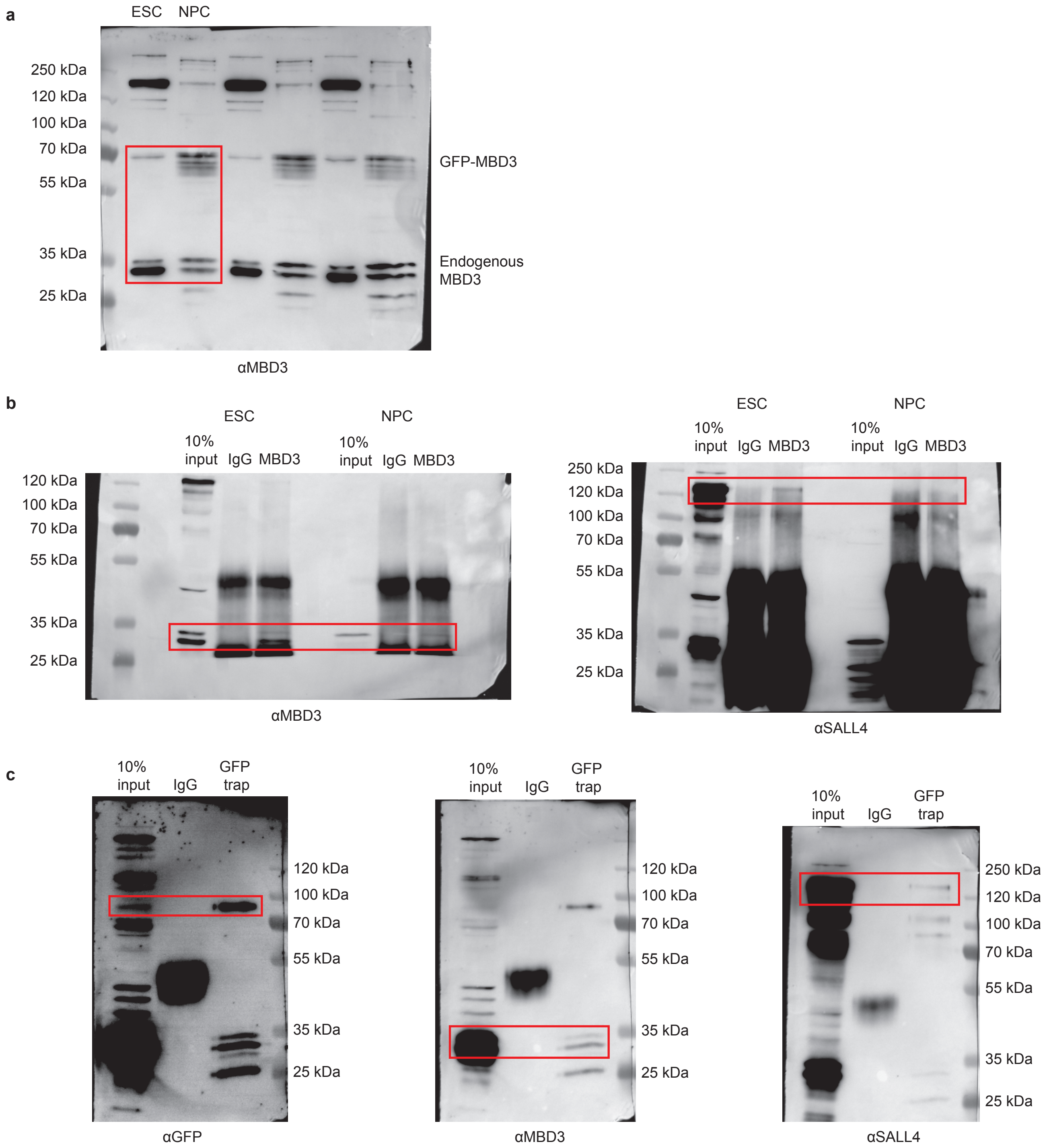

Supplementary Fig. 4: Uncropped Western blots.
(a) Uncropped Western blot corresponding to Supplementary Fig. 2a.
(b) Uncropped Western blots corresponding to Supplementary Fig. 2d.
(c) Uncropped Western blots corresponding to Supplementary Fig. 2g.

Target		Sequence (5'-3')
Nanog	For	CTTGACCTGAAACTTCCCACTA
	Rev	GGGCATCCTCTGATCTAAAGAC
Esrrb	For	CCTTGAACTGTTGCTCCTTCT
	Rev	GCTCAACTTTGCAGTTGAATACC
Pou5f1	For	GACGGCAGATGCATAACAAAG
	Rev	GCAGATTAAGGAAGGGCTAGG
Sall4	For	GCGAGGAGCGCTAAATTACTA
	Rev	CCAAATGCCCTTTGTGCTAAA
Tbx3	For	GTTCTGAGGTCACGGGTTTAG
	Rev	CAGAAAGACCAGCTGCTTAGA
Ubtd1	For	CCACACAGTCTAGCAGCTATTG
	Rev	CAACTGGAACTGTGCACAAATAA
Notch1	For	CAACATCTGGTTGGTGTTTGT
	Rev	CAGTTCTCACAGCCCTAGAAG
Smad3	For	CCCAGTACAACAGAGGAAGTTAG
	Rev	GATGTTGCTAAGAGTGTTGTGAAA
Atf3	For	CAATCCCAGGCTGACGTAAT
	Rev	TCGCACTTGCATCACCAA
Yap1	For	TCCTCGACGCTCTTCCTT
	Rev	GGGAGTCTGCAGGAATGTAG

Supplementary Table 1: Primers used for ChIP-qPCR analysis in this study.

Target		Sequence (5'-3')
Lefty2	For	CAGCCAGAATTTTCGAGAGGT
	Rev	CAGTGCGATTGGAGCCATC
Dazl	For	ATGTCTGCCACAACTTCTGAG
	Rev	CTGATTTCGGTTTCATCCATCCT
Pou5f1	For	TTGAGAACCGTGTGAGGTGG
	Rev	TCGGGCACTTCAGAAACATG
Rex1	For	ACGAGTGGCAGTTTCTTCTTGGGA
	Rev	TATGACTCACTTCCAGGGGGCACT
Fgf5	For	CATCTTCTGCAGCCACCTGATCCA
	Rev	AAGTTCCGGTTGCTCGGACTGCTT
Nestin	For	GCCTATAGTTCAACGCCCCC
	Rev	AGACAGGCAGGGCTAGCAAG
Otx2	For	TATCTAAAGCAACCGCCTTACG
	Rev	AAGTCCATACCCGAAGTGGTC
Nkx2.2	For	AAGCATTTCAAAACCGACGGA
	Rev	CCTCAAATCCACAGATGACCAGA
Tbx6	For	ATGTACCATCCACGAGAGTTGT
	Rev	GGTAGCGGTAACCCTCTGTC
Bmp2	For	GGGACCCGCTGTCTTCTAGT
	Rev	TCAACTCAAATTCGCTGAGGAC
Nodal	For	AGACGTTCACCGTCATTCCTT
	Rev	CCAACACTTTTCTGCTCGACT
Foxa2	For	GGAGGCAAGAAGACCGCTC
	Rev	CCTTTAGCTCGCTTAGGCCAC
Sox17	For	GATGCGGGATACGCCAGTG
	Rev	CCACCACCTCGCCTTTCAC
Gata4	For	CCCTACCCAGCCTACATGG
	Rev	ACATATCGAGATTGGGGTGTCT
Gapdh	For	TTCATTGACCTCAACTACATG
	Rev	GTGGCAGTGATGGCATGGAC
Actb	For	GGCTGTATTCCCCTCCATCG
	Rev	CCAGTTGGTAACAATGCCATGT

Supplementary Table 2: Primers used for qRT-PCR analysis in this study.

Name	Application	Supplier	Product number
Rabbit polyclonal anti-MBD3	ChIP-seq	Bethyl Laboratories	A302-528A
Mouse monoclonal anti-MBD3	IP, WB	IBL	JP10281
Mouse monoclonal anti-CHD4	ChIP-seq	abcam	ab70469
Rabbit polyclonal anti-SALL4	WB	abcam	ab29112
Rabbit polyclonal anti-GFP	ChIP-seq	abcam	ab290
Mouse monoclonal anti-GFP	WB	Roche	11814460001
Rabbit polyclonal anti-SIN3A	ChIP-seq	abcam	ab3479
Rabbit polyclonal anti-H3K27ac	ChIP-seq	Diagenode	A1723-0041d
Rabbit polyclonal anti-H3K4me3	ChIP-seq	Diagenode	A5051-0001P
Rabbit polyclonal anti-H3K4me1	ChIP-seq	Diagenode	A1863-001P
Rabbit polyclonal anti-H3K27me3	ChIP-seq	Diagenode	A1811-0001P

Supplementary Table 3: Antibodies used in this study.

Supplementary References

56. Stadler, M. B. *et al.* DNA-binding factors shape the mouse methylome at distal regulatory regions. *Nature* 480, 490–5 (2011).
