# Supplementary material for: NuRD-interacting protein ZFP296 regulates genome-wide NuRD localization and differentiation of mouse embryonic stem cells
Source: Nat Commun. 2018 Nov 2;9:4588. doi: 10.1038/s41467-018-07063-7 (PMC6214896; doi:10.1038/s41467-018-07063-7)
Supplement: Supplementary file 3 — Description of Additional Supplementary Files [file 41467_2018_7063_MOESM3_ESM.pdf]

Description of Additional Supplementary Files

File Name: Supplementary Data 1
Description: List of genes nearby ChIP-seq peaks

File Name: Supplementary Data 2
Description: iBAQ values of MBD3-interacting proteins.

File Name: Supplementary Data 3
Description: Differentially expressed genes and proteins in Zfp296 KO ESC.
